# Phylogeny of Cortical‐Hippocampal Projections Reveals Selective Elimination of Input From Sensory Regions

**DOI:** 10.1002/hipo.70119

**Published:** 2026-09-03

**Authors:** Daniel Reznik, Piotr Majka, Marcello G. P. Rosa, Menno P. Witter, Christian F. Doeller

**Affiliations:** ^1^ Kavli Institute for Systems Neuroscience, Centre for Algorithms in the Cortex, NTNU Trondheim Norway; ^2^ Oxford Centre for Integrative Neuroimaging University of Oxford Oxford UK; ^3^ Laboratory of Neuroinformatics Nencki Institute of Experimental Biology of the Polish Academy of Sciences Warsaw Poland; ^4^ Department of Physiology and Neuroscience Program Biomedicine Discovery Institute, Monash University Melbourne Australia; ^5^ Kavli Institute for Systems Neuroscience, Norwegian Health Association Centre for Dementia Research, NTNU Trondheim Norway

**Keywords:** connectivity, cortical organization, episodic memory, evolution, hippocampus, medial temporal lobe

## Abstract

The study of cross‐species differences in the organization of the cerebral cortex received significant attention during the past century. However, the evolutionary principles governing changes in brain connectivity and their impact on cognition remain poorly understood. In the current study, we analyzed differences in anatomical input from the cerebral cortex to the hippocampal region across six species spanning more than 100 million years of mammalian evolution—the tenrec, rat, cat, marmoset, macaque and human. Our results demonstrate that, unlike direct transmodal cortical input, direct unimodal cortical input to the hippocampal region was selectively eliminated with the increase in brain size. Additionally, input from primary sensory regions was eliminated prior to input from non‐primary sensory regions. Our findings suggest that hippocampus‐related processes in different species operate on fundamentally different types of information, potentially underpinning cross‐species differences in hippocampus‐dependent cognition. Furthermore, our findings outline the evolutionary trajectory of the anatomical circuitry associating the human hippocampal region with the neocortex.

## 1 Introduction

Cross‐species differences in cognition are typically attributed to the changes that occurred to the cerebral cortex through evolution (Krubitzer 2007, 2009; Reardon et al. 2018). This includes changes in size, structural differentiation and functional specialization of the cortex into an increasingly larger number of cortical areas, which have been hypothesized to enable an increase in the animal's behavioral range, its flexibility and complexity (Kaas 1989). Comparative research into the mammalian lineage has demonstrated that even though the evolution of the cerebral cortex followed an ancient phylogenetic template, the relative size of cortical areas involved in non‐sensory processing dramatically increased through mammalian evolution, especially in humans, playing a major role in the development of unprecedented high‐order cognitive functions (Buckner 2026; Buckner and Krienen 2013; Krubitzer 2009; Smaers et al. 2021). Over the past century, significant attention has been devoted to the study of differences in the organization of the cortex across species, and to the potential contribution of this factor to cross‐species differences in cognition (Brodmann 1909; Buckner and Krienen 2013; Chaplin et al. 2013; Kaas 1989; Krubitzer 2007; Orban et al. 2004; Petersen et al. 2024; Rosa and Manger 2005; Smaers et al. 2021). However, the notion that animal behavior is the product of coordinated activity between anatomically interconnected brain regions (Bargmann and Marder 2013; Goldman‐Rakic 1988) was rarely addressed from a comparative perspective. Hence, the evolutionary principles associating changes in anatomical connectivity with cross‐species variations in cognition remain poorly understood.

Anatomical studies in mammals indicate that whole‐brain cortical connectivity converges into the hippocampal region, comprising the hippocampal formation, perirhinal cortex, entorhinal cortex and parahippocampal cortex (postrhinal in the rodent) (Felleman and Van Essen 1991; Witter et al. 1989). Consequently, the hippocampal region is critically implicated in cognitive functions requiring large‐scale multimodal integration, such as memory, navigation, future planning and social cognition (Allen and Fortin 2013; Eichenbaum 2000, 2017; Manns and Eichenbaum 2006). The complex nature of intrinsic anatomical organization and connections of the hippocampal region provided multiple opportunities for phenotypical divergence across mammals. However, in striking contrast to the cerebral cortex, the gross anatomical, cytoarchitectural and functional properties of the hippocampal region have remained largely conserved during 200 million years of mammalian evolution (Amaral and Witter 1989; Manns and Eichenbaum 2006; Witter et al. 1989). This remarkable preservation of the hippocampal region across species is consistent with its role as a major connectivity anchor positioned at the pinnacle of the distributed cortical hierarchy (Burwell 2000; Felleman and Van Essen 1991; Reznik et al. 2024). These anatomical and functional properties of the hippocampal region raise the possibility that it performs similar intrinsic computations across species, albeit operating on different types of cortical input (Kaas 2019; Manns and Eichenbaum 2006), which could potentially contribute to cross‐species differences in hippocampus‐dependent functions (Jeffery et al. 2024; Martinez‐Trujillo 2025; Rolls 1999). This notion received preliminary support from comparing cortical connectivity of the hippocampal region across rats and macaques (Burwell et al. 1995), and across macaques and humans (Eichert et al. 2024; Reznik et al. 2023), suggesting that some connectivity patterns are preserved, while others have changed across species (Assaf et al. 2020; Herculano‐Houzel et al. 2010; Ringo 1991; Suárez et al. 2018).

**FIGURE 1:**
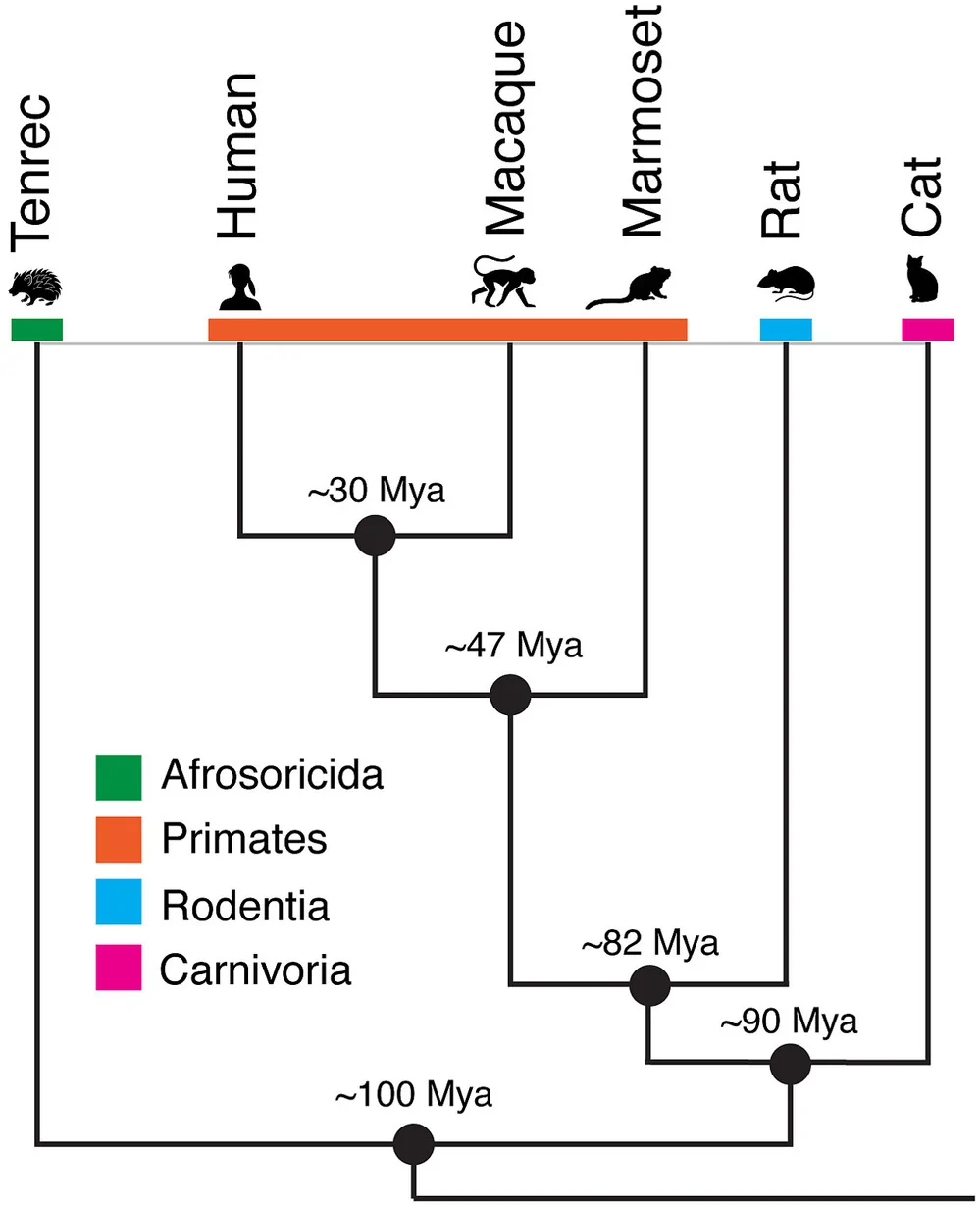
Mammalian species used in this study. We analyzed tract‐tracing data from the tenrec, rat, cat, marmoset and macaque, which cover around 100 million years of mammalian evolution. Note that despite long periods of independent evolution, tenrecs and rats have brains with relatively similar sizes, whereas the marmoset split from the common ancestor with the macaque relatively recently but has a much smaller and smoother brain compared with macaques (Heuer et al. 2025).

In the current study, we leveraged the contrast between the preserved hippocampal region and the diverse cortex to test whether the type of information funneled into the hippocampal region systematically changed through evolution. To this end, we examined differences in cortical input to the hippocampal region from unimodal and transmodal cortical areas across all mammalian species with known connectivity of the hippocampal region—the tenrec ( *Echinops telfairi* ), rat ( *Rattus norvegicus* ), cat ( *Felis catus* ), marmoset ( *Callithrix jacchus* ), macaque ( *Macaca fascicularis* ), and human ( *Homo sapiens* ). These six species represent branches of the mammalian evolutionary tree that diverged over more than 100 million years of evolution (Figure 1). We analyzed anatomical tract‐tracing data for the tenrec, rat, cat, marmoset and macaque, and functional magnetic resonance imaging (fMRI) data for humans as a proxy of anatomical connectivity (Liu et al. 2019; Vincent et al. 2007, 2010). Our analyses point to gradual elimination of direct anatomical input from unimodal sensory regions and selective preservation of direct input from transmodal cortical regions, thus potentially outlining a major trend in the evolution of cortico‐hippocampal circuitry during mammalian phylogeny.

## 2 Materials and Methods

The central aim of this study was to provide a cross‐species comparative framework to the changes in anatomical connectivity associating the mammalian cortex with the hippocampal region. Due to the dramatic differences in the structural properties of the cortex across species, it is a challenging task to define homologous cortical regions that are present in all species, especially when cortical regions involved in non‐sensory processing are considered (Brodmann 1909; Rosa and Manger 2005; Uylings et al. 2003). Therefore, we avoided comparing input from potential cortical homologues across species; instead, we utilized the phylogenetically conserved property of cortical hierarchy and divided the cortex of each species into cortical regions involved in unimodal sensory processing and cortical regions involved in transmodal processing. Unimodal cortical areas were defined as areas that were shown to selectively respond predominantly to one type of sensory modality, which include piriform, visual, auditory and somatosensory cortices. Where possible, unimodal sensory cortical regions were divided into primary sensory regions and non‐primary sensory regions. Primary sensory regions, with the exception of the piriform cortex, were defined by direct input from sensory thalamic nuclei; non‐primary sensory regions were defined by lack of connections with primary thalamic nuclei and by input from primary sensory regions. Transmodal cortical regions were defined as regions that do not show specificity to any single sensory modality, which included polymodal and limbic cortical regions (Mesulam 1998). See Supporting Information for more details about the mapping of unimodal and transmodal regions in each species.

In the tenrec, we used data from studies that examined anatomical connections of the tenrec hippocampal region (Künzle 2002, 2003). In the rat, we used a prior analysis of more than 16,000 connectivity reports, including the hippocampal region (Bota et al. 2015), as well as data from a study that examined input to the hippocampal region using retrograde tracer injections (Burwell and Amaral 1998). In the marmoset, we used an online brain connectivity atlas, which summarizes data from multiple fluorescent retrograde tracer injections www.marmosetbrain.org; (Majka et al. 2016, 2020). Additionally, we used connectivity data that are not yet publicly available and will be added to the marmoset online atlas in future releases. In the cat, similar to the rat, we used a previous analysis of cortical connectivity, including the hippocampal region, derived from more than 90 anatomical connectivity studies (Scannell et al. 1995), as well as data from a study that examined connectivity between the cat's prepiriform cortex and entorhinal cortex (Krettek and Price 1977). In the macaque, we used studies that examined input to the hippocampal region using retrograde tracer injections, as well as studies that examined output projections of distributed cortical regions, including to the hippocampal region, using anterograde tracer injections (Cavada and Goldman‐Rakic 1989; Ding et al. 2000; Insausti et al. 1987; Lavenex et al. 2002; Mohedano‐Moriano et al. 2007; Muñoz and Insausti 2005; Seltzer and Pandya 1984; Suzuki and Amaral 1994). In humans, we used recent precision fMRI connectivity studies that examined the functional architecture of the hippocampal region at the individual‐subject level (Angeli et al. 2025; Edmonds et al. 2024; Reznik et al. 2023, 2024; Zheng et al. 2021).

First, in each species, we calculated the proportion of the cortical mantle dedicated to unimodal sensory processing and to transmodal processing (Table S1–S6). To this end, previous cortical parcellations were used for each species. Next, using existing tract‐tracing (for non‐human animals) and fMRI connectivity (for humans) data, we calculated the overall proportion of the cortex, collapsed across unimodal and transmodal regions, projecting to the hippocampal region (or associated with it, in the case of fMRI). Connectivity was considered for each separate cortical region in each species. Since tracer injections or labeled cells often target a portion of different subregions, the entire subregion was considered to have connections with the hippocampal region even if only a portion was examined or showed labeled cells.

Lastly, in each species, we calculated separately the proportion of unimodal and transmodal regions projecting to the hippocampal region. Importantly, whereas in the tenrec, cat, rat, and macaque, the anatomical projections to the hippocampal region were estimated directly by examining labelling of cells following tracer injections into the hippocampal region or distributed cortical regions, in the marmoset and human, cortical projections to the hippocampal region were estimated indirectly (see Supporting Information for more details).

To account for methodological differences in quantification of animal tracing data, we included all anatomical connections, including weak cortical input to the hippocampal region (e.g., 1%–5% connectivity in the macaque; connections that were originally described as occasional or sporadic were not included in the analysis). In the tenrec, since the only studies that reported anatomical connectivity of the hippocampal region used a subjective rating scale (Künzle 2002, 2003), only connections that were rated 3 (moderate connectivity) or above were included in the analysis. In the marmoset, since only data on anatomical projections from the hippocampal region are available (Majka et al. 2020), anatomical projections to the hippocampal region were estimated based on the reciprocity of the anatomical connections between the hippocampal region and the broader neocortex in the macaque (Lavenex et al. 2002). To account for weaker return projections from the hippocampal region to the primate cortex (Lavenex et al. 2002), we used a connectivity threshold of log_10_(FLNe) = −3 (fraction of extrinsic labeled neurons; average value across all available experiments in the same cortical area) in the marmoset. Only connections that showed up in more than half of the experiments for each cortical area in the marmoset were considered. For calculating the final estimate of marmoset projections from the neocortex to the hippocampal region, we averaged the percentage of input projections estimated solely on output projections and the percentage of input projections estimated using anatomical data in the macaque (see Supporting Information for more details, including Figure S1 for analysis without macaque‐based adjustment of marmoset data).

For interpreting the associations of the human hippocampal region with the cerebral cortex estimated with fMRI connectivity, we used a network‐based approach, meaning that only distributed networks that were previously reported to associate with the human hippocampal region were included in the analysis (but see Bergmann et al. 2016 and Wang et al. 2016). It is important to emphasize that human connectivity data should be interpreted in light of our methodological choice to focus on fMRI connectivity findings. Other non‐invasive methods, such as diffusion‐weighted imaging (DWI), provide as well important insights into the anatomical organization of the human hippocampal region (Dalton et al. 2022; Huang et al. 2021; Maller et al. 2019). Specifically, while fMRI connectivity did not reveal associations between the human hippocampal region and early visual areas (Reznik et al. 2023), a recent DWI connectivity study points to such visual‐hippocampal associations (Dalton et al. 2022).

Following recent findings demonstrating that animals with similar brain sizes show similar connectivity and behavioral phenotypes, irrespective of their phylogenetic similarity (Heuer et al. 2025), we examined how changes in cortical input to the hippocampal region are associated with species' cortical surface area (log‐transformed square millimeters), without taking phylogenetic relationships into account. We used previous studies to estimate cortical surface area in the tenrec (Krubitzer et al. 1997), rat (Ventura‐Antunes et al. 2013), marmoset (Majka et al. 2016), cat (Rathjen et al. 2003), macaque (Felleman and Van Essen 1991), and human (Donahue et al. 2018).

## 3 Results

**FIGURE 2:**
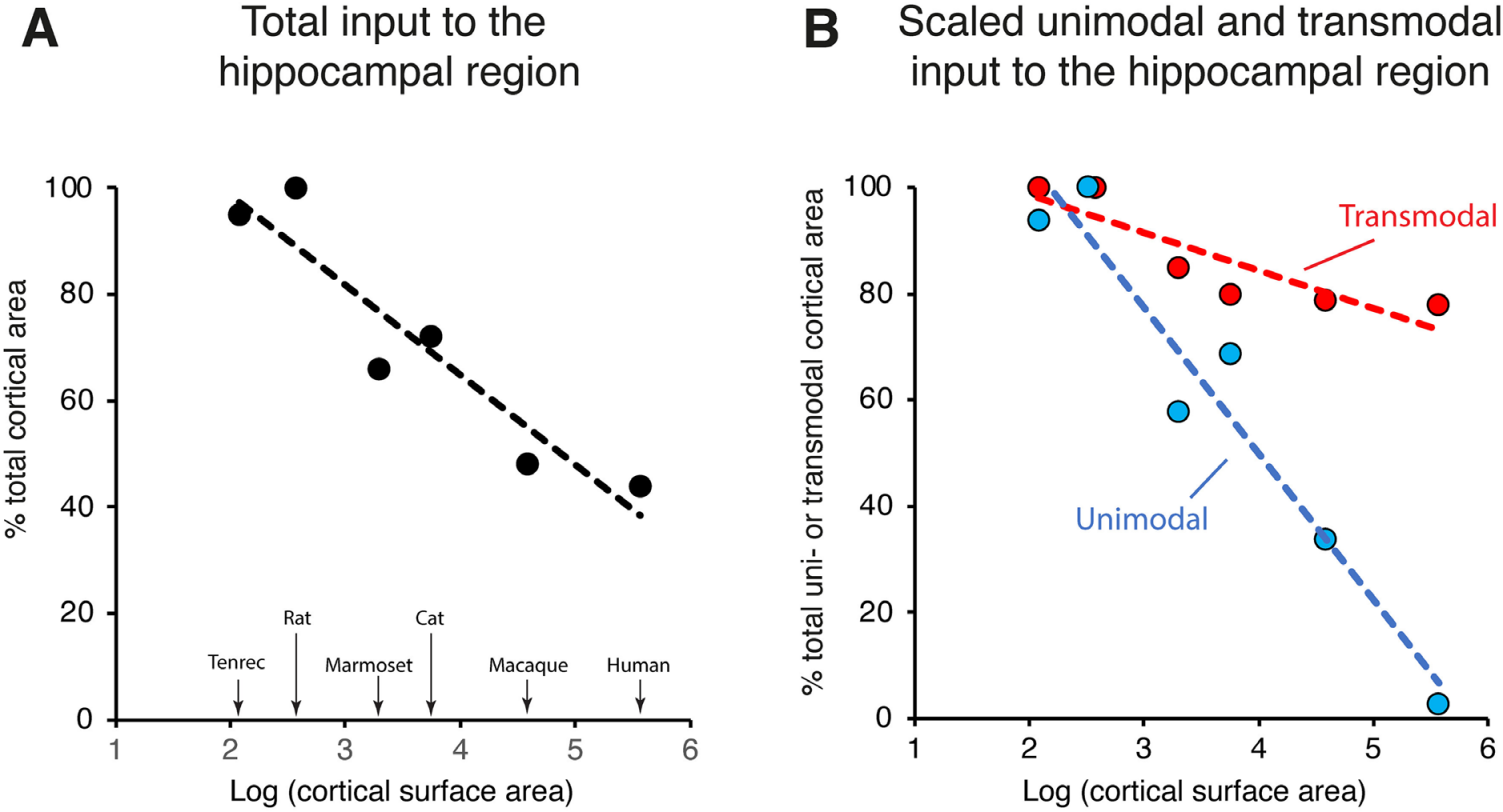
Cortical input to the hippocampal region. (A) We find that the percentage of total cortical area (collapsed across unimodal and transmodal cortices) that projects to the hippocampal region decreases across species with the increase in brain size (Pearson correlation coefficient *r* = −0.92, *p* < 0.01). In small brain mammals (the tenrec and rat), almost the entire cortical mantle projects directly to the hippocampal region. In primates, about half of the cortical mantle projects directly to the hippocampal region. (B) Percentage of cortical input to the hippocampal region calculated separately from the total unimodal and transmodal cortical areas in each species. This analysis demonstrates that even though there is a general decrease in the percentage of total cortical area projecting to the hippocampal region, this decrease disproportionally targets unimodal input, while input from transmodal cortical areas remains relatively preserved with the increase of brain size (repeated measures ANCOVA, *p* < 0.01).

We examined cross‐species changes in anatomical projections from the cortex to the hippocampal region in six different species (Figure 1). Our results indicate that the percentage of total cortical area projecting to the hippocampal region reduces with the increase in cortical surface area (Figure 2A; Pearson correlation coefficient = −0.94, *p* < 0.01). In small non‐primate mammals—the tenrec and rat—nearly the entire cortical mantle directly projects to the hippocampal region. However, in the marmoset, macaque and human, cortical subregions corresponding to about half of the cortex project to the hippocampal region. Next, we calculated direct input to the hippocampal region from unimodal and transmodal cortical regions relative to the total unimodal and transmodal cortical area in each species. We observed that relative input from each modality reduced with the increase in cortical surface area. Critically, this reduction disproportionally targeted input from unimodal cortical areas, while the input from transmodal areas was relatively preserved across species (Figure 2B; repeated measures ANCOVA, F_(1,4)_ = 22.84, *p* < 0.01; see Figure S2 for unimodal and transmodal input relative to total cortical surface; see Figure S3 for an analysis using only tract tracing data, excluding humans). For example, in the rat, all primary and non‐primary sensory regions have monosynaptic input to the hippocampal region. On the other hand, in larger mammals, such as cat and primates, we observed a gradual reduction in direct cortical input from unimodal sensory regions. Importantly, these changes in connectivity could not be explained merely by changes in the relative size of transmodal areas in different species. Even though about 1/3 of the human brain is involved in unimodal sensory processing, only the piriform cortex might be associated with the hippocampal region (see Discussion).

Intriguingly, when we examined the reduction of input from unimodal sensory regions separately for each sensory modality, two phenomena were observed. First, direct anatomical input from sensory regions was eliminated gradually across species. Second, elimination of anatomical input from primary sensory regions was followed by elimination of anatomical input from non‐primary sensory regions. Namely, in the rat, all primary and non‐primary sensory regions project to the hippocampal region. However, in the cat, projections from primary somatosensory regions are not present, while projections from non‐primary somatosensory, as well as from primary and non‐primary auditory and visual regions remain. In the macaque, projections from primary auditory and visual regions (which are present in the cat) are absent, leaving only projections from non‐primary auditory (caudal STG), non‐primary visual (areas V4, IT cortex), and non‐primary somatosensory (SII, granular insula) regions. The process of gradual elimination of anatomical connectivity with sensory regions culminates in humans, where connections with non‐primary sensory regions that are present in the macaque are further eliminated. One notable exception to this pattern is connections of the hippocampal region with the piriform cortex (see Discussion).

## 4 Discussion

In the current study we explored the evolutionary principles governing changes in anatomical connectivity between the cerebral cortex and the hippocampal region. To this end, we examined anatomical data from six mammalian species in which anatomical connections of the hippocampal regions are known. Our results point to a global reduction in cortical input to the hippocampal region with the increase in brain size. Critically, this reduction selectively targeted anatomical input from unimodal sensory regions, while input from transmodal cortical regions was relatively preserved. Furthermore, we observed that the elimination of unimodal sensory input was highly structured, such that direct input from primary sensory regions was eliminated first, followed by elimination of direct input from non‐primary sensory regions.

Previous cross‐species examinations of anatomical connectivity demonstrated that direct connections between neurons throughout the cortex are reduced with the increase in brain size, leading to the progressive segregation of the cortical circuitry into subnetworks (Herculano‐Houzel et al. 2010; Palmer and Rosa 2006; Ringo 1991; Rosa et al. 2019). This progressive disconnection potentially reflects the necessity to balance between the number of neurons and efficient information processing, as well as intrinsic limitations dictated by available membrane space for synapses (Luppi et al. 2024; Stevens 1989). The overall reduction in neural connectivity may be associated with the necessity to regulate the total amount of energy consumed by the brain, where most of the consumed energy is used for synaptic transmission (Harris et al. 2012). Additionally, most long‐range anatomical projections in studied mammals are myelinated (Liewald et al. 2014), which imposes additional connectivity‐related high energetic costs necessary for myelin synthesis and oligodendrocytes maintenance (Harris and Attwell 2012; Saab et al. 2013). However, our results indicate that at least when connectivity with the hippocampal region is considered, the global reduction in neural connectivity selectively targets connections with primary and non‐primary sensory regions. Specifically, our results show that the changes in cortical input to the hippocampal region reflect a continuous transition—from nearly all primary and non‐primary sensory regions that project to the hippocampal region in small‐brained mammals—the tenrec and rat—through only non‐primary auditory, visual and somatosensory areas that project to the hippocampal region in the macaque, culminating in humans, which show no connectivity with any of the non‐primary sensory regions.

While we currently do not have an answer to why specifically connections with unimodal sensory regions are eliminated, the observed trend is dramatic and indicates that the hippocampus operates on fundamentally different types of information in different species. Since at least some of the connections between the hippocampal region and the parietal, retrosplenial, cingulate, and limbic cortices observed in humans exist already in small‐brained mammals, we suggest that the gradual elimination of unimodal input through mammalian evolution reflects selective preservation of anatomical connectivity favoring input from transmodal regions.

One notable exception to the elimination of direct sensory input to the hippocampal region is input from the piriform cortex. Unlike other sensory modalities that were gradually disconnected from the hippocampal region, the piriform cortex directly projects to the entorhinal cortex in the rat, cat and macaque despite dramatic differences in brain size and cortical complexity across species. While there is no evidence showing direct projections from the piriform cortex to the hippocampal region in the tenrec (personal correspondence with Heinz Künzle), it is plausible that these connections exist based on anatomical data from other species. In humans, the piriform cortex is typically assigned to the limbic network (Yeo et al. 2011), which may be part of the canonical default network (Girn et al. 2024). Associations betweeh the human hippocampal region and subdivisions of the canonical default network suggest that such connections may exist in humans as well (Angeli et al. 2025; Reznik et al. 2023, 2024; Zheng et al. 2021). One possible explanation for the preserved connectivity with the piriform cortex is that, unlike other primary sensory cortices, which do not share a direct anatomical boundary with the hippocampal region, the piriform cortex is anatomically adjacent to and developmentally associated with the perirhinal and entorhinal cortex. Taken to the extreme, in the tenrec, the anatomical boundary between the piriform cortex and the entorhinal cortex is not clearly defined (Künzle and Radtke‐Schuller 2000). With the dramatic increase in brain size across species, the anatomical proximity of the piriform cortex barely changed, and it remained positioned closely to the perirhinal and entorhinal cortex, thus potentially retaining its anatomical connectivity with the hippocampal region.

It is intriguing to consider potential functional implications of such differences in cortical input to the hippocampal regions across species. In the early levels of cortical processing, primary and non‐primary sensory cortices encode different features of sensory information originating in the extrapersonal environment. In contrast, at later levels of cortical processing, transmodal areas converge these low‐level, modality‐specific signals and form cross‐modal associative representations. For example, the ventral stream of visual processing, beginning in primary visual cortex, transforms low‐level visual features into increasingly complex transmodal representations in anterior temporal cortex (Mishkin and Ungerleider 1982). Therefore, transmodal areas that are associated with the hippocampal region convey to the hippocampal region highly processed multisensory information which is not tuned to a single stimulus property, such as the size of the respective representation on the retina or sound frequency. Our findings imply that the synaptic distance between sensory perception and mnemonic encoding dramatically differs across species, which could be reflected in cross‐species differences in memory‐related behaviors. The hippocampal region in the broad sense is known to be involved in episodic memory, learning and navigation (Eichenbaum 2017; Igarashi et al. 2014; O'Keefe and Nadel 1978). These seemingly distinct functional properties of the hippocampus are typically considered separately and species‐specific. However, they can also be seen in tandem, representing a functional continuum of a hierarchical increase in mnemonic complexity across species (Eichenbaum 2017; Jeffery et al. 2024). For example, early memory theories suggested an existence of multiple memory systems that can be classified, for example, into “associative memory,” “representational memory” and “abstract memory” (Oakley 1981). Importantly, these systems were proposed to be phylogenetically dependent on each other, such that different systems emerged at different stages of animal evolution (Tulving 1985). More recent accounts of spatial navigation suggest a common framework that can account for the rich behavioral and experimental variability across different species (Jeffery et al. 2024). According to this framework, human memory can be studied as a form of navigation albeit in abstract spaces (Bellmund et al. 2018), even though these two allegedly distinct functions are typically tested by different experimental paradigms.

**FIGURE 3:**
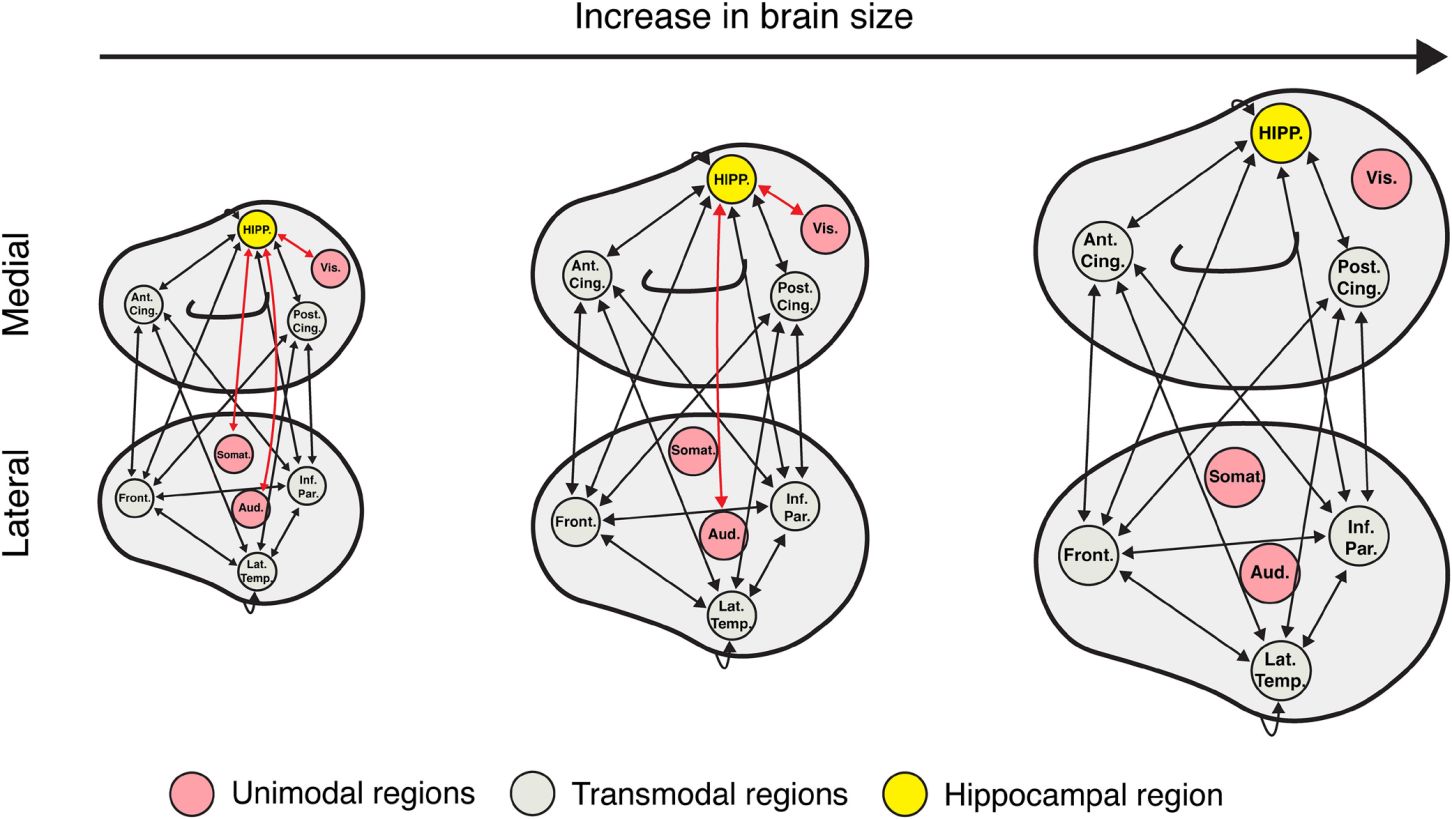
A schematic model describing the evolutionary trajectory of hippocampal circuitry. In small brain mammals, nearly all cortical regions project to the hippocampal region (denoted in the figure as HIPP). With the increase in brain size, direct projections from unimodal regions (red) gradually disappear, but direct projections from transmodal regions (gray) remain. When the cortical size reaches the level of human cortex, no connections between the hippocampal region and unimodal regions exist, and the hippocampal circuitry is almost exclusively associated with transmodal cortical regions. Ant. Cing.—Anterior cingulate; Aud.—Auditory cortex; Inf. Par.—Inferior parietal cortex; Lat. Temp.—Lateral temporal cortex; Post. Cing.—Posterior cingulate cortex; Somat.—Somatosensory cortex; Vis.—Visual cortex.

Our current results suggest that the notion of memory may have changed through evolution from supporting sensory‐based functions, such as response inhibition (O'Keefe and Nadel 1978), to supporting sensory‐detached functions, such as episodic construction, future thinking and mental time travel (Buckner and Carroll 2007; Hassabis and Maguire 2007). It is difficult to imagine mnemonic processing driven by low‐level sensory features (e.g., in the tenrec or rat); nevertheless, animal studies show that marmoset monkeys perform better in visual memory tasks requiring object‐centred perception compared with rats, which are more prone to viewer‐centred bias (Kell et al. 2023). Even though it is challenging to compare visual perception across species due to dramatic differences in the organization of the visual system ‐ for example, rats have a very wide field of view and lack a fovea (De Araujo et al. 2001; Solomon and Rosa 2014) ‐ this finding is consistent with reduced visual‐mnemonic hierarchy in the rat compared with the marmoset (Wang et al. 2020), suggesting that in rats, mnemonic fidelity to low‐level sensory features potentially hinders object‐centred generalization. We postulate that the transition from sensory‐based mnemonic cognition to increasingly abstract cognition (Tolman 1948) is associated with the increasingly dominating role of transmodal input to the hippocampal region. Intriguingly, the distributed circuitry between transmodal cortical regions and its detachment from sensory processing was previously proposed to be especially expanded in humans and suited especially well for “internal mentation” functions, with some of them being highly dependent of the hippocampus, such as episodic recall, imagery and future thinking (Buckner and Krienen 2013). Our current findings outline the potential evolutionary trajectory underlying the development of this circuitry into the human form (Figure 3).

The current study has three main limitations. The first limitation is our sample size. We analyzed data from only six species—the only species with known distributed connectivity of the hippocampal region—four species representing Euarchontoglires (the rat, marmoset, macaque and human), one species representing Afrotheria (the tenrec) and one species representing Laurasiatheria (the cat). This narrow sample size limited our ability to examine broad cross‐taxa associations. Therefore, we are cautious in claiming that the observed results represent a general principle in mammalian phylogeny. For example, certain mammals, such as cetaceans, show a remarkably underdeveloped hippocampal region (Patzke et al. 2015), suggesting that at least some mammalian species might use other mechanisms for memory‐related tasks. The second limitation is the indirect estimation of anatomical connectivity in humans using fMRI connectivity methods. Even though these methods were proved to be valid tools for non‐invasively studying neuroanatomical connectivity (Vincent et al. 2007), unlike tract‐tracing methods in the tenrec, cat, rat, marmoset and macaque, they are a proxy for measuring neuroanatomical connections. Therefore, absence of functional connectivity between brain regions in humans cannot indicate lack of anatomical connectivity. Nevertheless, a similar limitation holds also for animal tracing experiments, which often contain “hidden” data, either unpublished or not looked for. Therefore, no evidence for anatomical connectivity between certain regions in animal studies does not mean that this connectivity does not exist. The third limitation of our study is the definition of unimodal and transmodal cortical areas, which is particularly challenging given evidence pointing to multisensory properties of early sensory cortices (Schroeder and Foxe 2005). However, since the functional properties of low‐level multisensory integration are not yet clarified and their anatomical and physiological profiles contrast with those in “classic” multisensory areas (e.g., ventral intraparietal sulcus and superior temporal sulcus) (Smiley et al. 2007), in the current study we treated early sensory cortices as unimodal. Moreover, we adopted a neuroanatomical definition of unimodal sensory regions based on oligosynaptic access to information channeled by the sensory organs (e.g., retina, cochlea, tactile receptors).

To conclude, our study provides preliminary evidence for a continuous trend towards an increased role of transmodal input in mnemonic processing during 100 million years of mammalian evolution culminating in humans. Our findings suggest that the hippocampal region operates on fundamentally different types of input in different species, which would lead to specific hypotheses addressing differences in mnemonic representations in different species. Finally, our study suggests that it is critical to adopt an evolutionary perspective into the study of the hippocampal region, especially when cross‐species data are considered (Jeffery et al. 2024; Martinez‐Trujillo 2025; Quian Quiroga 2023; Zhu et al. 2023).

## Author Contributions

D.R.: conceptualization, formal analysis, writing – original draft preparation, reviewing and editing; P.M.: resources, writing – reviewing and editing; M.G.P.R.: resources, writing – reviewing and editing; M.P.W.: writing – reviewing and editing; C.F.D.: funding acquisition, writing – reviewing and editing. All authors read and approved the final version of the manuscript.

## Funding

This work was supported by the Max Planck Society, the Kavli Foundation. The K.G. Jebsen Centre for Alzheimer's Disease. Australian Research Council, DP210101042, DP210103865. National Health and Medical Research Council, APP1194206, APP2041019. National Science Centre Poland, 2019/35/D/NZ4/03031. European Research Council (ERC) under the European Union's Horizon Europe research and innovation programme, grant agreement 101220569.

## Supporting information


**Table S1:** Unimodal and transmodal cortical areas in the tenrec, rat and cat.
**Table S2:** Unimodal and transmodal cortical areas in the marmoset, macaque and human.
**Table S3:** Number of electrodes that showed different response patterns in tenrec neocortex.
**Table S4:** Number of voxels assigned to each cortical subdivision in the cat.
**Table S5:** Size (in mm^3^) of cortical subdivisions in the marmoset.
**Table S6:** Number of surface vertices assigned to each cortical network in the human.
**Figure S1:** Cortical input to the hippocampal region without adjusted marmoset data.
**Figure S2:** Unimodal and transmodal areas and total cortical input to the hippocampal region.
**Figure S3:** Cortical input to the hippocampal region without human data.

## Data Availability

The data that supports the findings of this study are available in the Supporting Information of this article.

## References

[hipo70119-bib-0001] Allen, T. A. , and N. J. Fortin . 2013. “The Evolution of Episodic Memory.” Proceedings of the National Academy of Sciences 110: 10379–10386. 10.1073/pnas.1301199110. PMC3690604 23754432

[hipo70119-bib-0002] Amaral, D. G. , and M. P. Witter . 1989. “The Three‐Dimensional Organization of the Hippocampal Formation: A Review of Anatomical Data.” Neuroscience 31, no. 3: 571–591. 10.1016/0306-4522(89)90424-7. 2687721

[hipo70119-bib-0004] Angeli, P. A. , L. M. DiNicola , N. Saadon‐Grosman , M. C. Eldaief , and R. L. Buckner . 2025. “Specialization of the Human Hippocampal Long Axis Revisited.” Proceedings of the National Academy of Sciences 122, no. 3: e2422083122. 10.1073/PNAS.2422083122. PMC11760929 39808662

[hipo70119-bib-0005] Assaf, Y. , A. Bouznach , O. Zomet , A. Marom , and Y. Yovel . 2020. “Conservation of Brain Connectivity and Wiring Across the Mammalian Class.” Nature Neuroscience 23, no. 7: 805–808. 10.1038/s41593-020-0641-7. 32514137

[hipo70119-bib-0006] Bargmann, C. I. , and E. Marder . 2013. “From the Connectome to Brain Function.” Nature Methods 10, no. 6: 483–490. 10.1038/nmeth.2451. 23866325

[hipo70119-bib-0007] Bellmund, J. L. S. , P. Gärdenfors , E. I. Moser , and C. F. Doeller . 2018. “Navigating Cognition: Spatial Codes for Human Thinking.” Science 362, no. 6415: eaat6766. 10.1126/science.aat6766. 30409861

[hipo70119-bib-0008] Bergmann, E. , G. Zur , G. Bershadsky , and I. Kahn . 2016. “The Organization of Mouse and Human Cortico‐Hippocampal Networks Estimated by Intrinsic Functional Connectivity.” Cerebral Cortex 26, no. 12: 4497–4512. 10.1093/cercor/bhw327. 27797832 PMC5193145

[hipo70119-bib-0009] Bota, M. , O. Sporns , and L. W. Swanson . 2015. “Architecture of the Cerebral Cortical Association Connectome Underlying Cognition.” Proceedings of the National Academy of Sciences of the United States of America 112, no. 16: E2093–E2101. 10.1073/PNAS.1504394112/SUPPL_FILE/PNAS.201504394SI.PDF. 25848037 PMC4413280

[hipo70119-bib-0010] Brodmann, K. 1909. Brodmann's Localization in the Cerebral Cortex, edited by L. J. Garvey . Springer.

[hipo70119-bib-0011] Buckner, R. L. 2026. “Topography of Cognition: Evolution of Parallel Distributed Association Networks in Primates.” In Evolution of Nervous Systems, 363–389. Elsevier. 10.1016/B978-0-443-27380-3.00009-9.

[hipo70119-bib-0012] Buckner, R. L. , and D. C. Carroll . 2007. “Self‐Projection and the Brain.” Trends in Cognitive Sciences 11, no. 2: 49–57. 10.1016/j.tics.2006.11.004. 17188554

[hipo70119-bib-0013] Buckner, R. L. , and F. M. Krienen . 2013. “The Evolution of Distributed Association Networks in the Human Brain.” Trends in Cognitive Sciences 17, no. 12: 648–665. 10.1016/j.tics.2013.09.017. 24210963

[hipo70119-bib-0014] Burwell, R. D. 2000. “The Parahippocampal Region: Corticocortical Connectivity.” Annals of the New York Academy of Sciences 911, no. 1: 25–42. 10.1111/j.1749-6632.2000.tb06717.x. 10911865

[hipo70119-bib-0015] Burwell, R. D. , and D. G. Amaral . 1998. “Cortical Afferents of the Perirhinal, Postrhinal, and Entorhinal Cortices of the Rat.” Journal of Comparative Neurology 398, no. 2: 179–205. 9700566 10.1002/(sici)1096-9861(19980824)398:2<179::aid-cne3>3.0.co;2-y

[hipo70119-bib-0016] Burwell, R. D. , M. P. Witter , and D. G. Amaral . 1995. “Perirhinal and Postrhinal Cortices of the Rat: A Review of the Neuroanatomical Literature and Comparison With Findings From the Monkey Brain.” Hippocampus 5, no. 5: 390–408. 10.1002/hipo.450050503. 8773253

[hipo70119-bib-0017] Cavada, C. , and P. S. Goldman‐Rakic . 1989. “Posterior Parietal Cortex in Rhesus Monkey: I. Parcellation of Areas Based on Distinctive Limbic and Sensory Corticocortical Connections.” Journal of Comparative Neurology 287, no. 4: 393–421. 10.1002/cne.902870402. 2477405

[hipo70119-bib-0018] Chaplin, T. A. , H.‐H. Yu , J. G. M. Soares , R. Gattass , and M. G. P. Rosa . 2013. “A Conserved Pattern of Differential Expansion of Cortical Areas in Simian Primates.” Journal of Neuroscience 33, no. 38: 15120–15125. 10.1523/JNEUROSCI.2909-13.2013. 24048842 PMC6618405

[hipo70119-bib-0019] Dalton, M. A. , A. D'Souza , J. Lv , and F. Calamante . 2022. “New Insights Into Anatomical Connectivity Along the Anterior–Posterior Axis of the Human Hippocampus Using in Vivo Quantitative Fibre Tracking.” eLife 11. 10.7554/eLife.76143. PMC9643002 36345716

[hipo70119-bib-0020] De Araujo, I. E. T. , E. T. Rolls , and S. M. Stringer . 2001. “A View Model Which Accounts for the Spatial Fields of Hippocampal Primate Spatial View Cells and Rat Place Cells.” Hippocampus 11, no. 6: 699–706. 10.1002/hipo.1085. 11811664

[hipo70119-bib-0021] Ding, S. L. , G. Van Hoesen , and K. S. Rockland . 2000. “Inferior Parietal Lobule Projections to the Presubiculum and Neighboring Ventromedial Temporal Cortical Areas.” Journal of Comparative Neurology 425: 510–530. 10.1002/1096-9861(20001002)425:4<510::AID-CNE4>3.0.CO;2-R. 10975877

[hipo70119-bib-0022] Donahue, C. J. , M. F. Glasser , T. M. Preuss , J. K. Rilling , and D. C. Van Essen . 2018. “Quantitative Assessment of Prefrontal Cortex in Humans Relative to Nonhuman Primates.” Proceedings of the National Academy of Sciences of the United States of America 115, no. 22: E5183–E5192. 10.1073/pnas.1721653115. 29739891 PMC5984508

[hipo70119-bib-0023] Edmonds, D. , J. J. Salvo , N. Anderson , et al. 2024. “The Human Social Cognitive Network Contains Multiple Regions Within the Amygdala.” Science Advances 10, no. 47: 453. 10.1126/sciadv.adp0453. PMC11584017 39576857

[hipo70119-bib-0024] Eichenbaum, H. 2000. “A Cortical–Hippocampal System for Declarative Memory.” Nature Reviews Neuroscience 1, no. 1: 41–50. 10.1038/35036213. 11252767

[hipo70119-bib-0025] Eichenbaum, H. 2017. “The Role of the Hippocampus in Navigation Is Memory.” Journal of Neurophysiology 117: 1785–1796. 10.1152/jn.00005.2017.-There. 28148640 PMC5384971

[hipo70119-bib-0026] Eichert, N. , J. DeKraker , A. F. D. Howard , et al. 2024. “Hippocampal Connectivity Patterns Echo Macroscale Cortical Evolution in the Primate Brain.” Nature Communications 15, no. 1: 5963. 10.1038/s41467-024-49823-8. PMC11252401 39013855

[hipo70119-bib-0027] Felleman, D. J. , and D. C. Van Essen . 1991. “Distributed Hierarchical Processing in the Primate Cerebral Cortex.” Cerebral Cortex 1, no. 1: 1–47. 10.1093/cercor/1.1.1. 1822724

[hipo70119-bib-0028] Girn, M. , R. Setton , G. R. Turner , and R. N. Spreng . 2024. “The “Limbic Network,” Comprising Orbitofrontal and Anterior Temporal Cortex, Is Part of an Extended Default Network: Evidence From Multi‐Echo fMRI.” Network Neuroscience 8, no. 3: 860–882. 10.1162/netn_a_00385. 39355434 PMC11398723

[hipo70119-bib-0029] Goldman‐Rakic, P. 1988. “Topography of Cognition: Parallel Distributed Networks in Primate Association Cortex.” Annual Review of Neuroscience 11, no. 1: 137–156. 10.1146/annurev.neuro.11.1.137. 3284439

[hipo70119-bib-0030] Harris, J. J. , and D. Attwell . 2012. “The Energetics of CNS White Matter.” Journal of Neuroscience 32, no. 1: 356–371. 10.1523/JNEUROSCI.3430-11.2012. 22219296 PMC3272449

[hipo70119-bib-0031] Harris, J. J. , R. Jolivet , and D. Attwell . 2012. “Synaptic Energy Use and Supply.” Neuron 75, no. 5: 762–777. 10.1016/j.neuron.2012.08.019. 22958818

[hipo70119-bib-0032] Hassabis, D. , and E. A. Maguire . 2007. “Deconstructing Episodic Memory With Construction.” Trends in Cognitive Sciences 11, no. 7: 299–306. 10.1016/j.tics.2007.05.001. 17548229

[hipo70119-bib-0033] Herculano‐Houzel, S. , B. Mota , P. Wong , and J. H. Kaas . 2010. “Connectivity‐Driven White Matter Scaling and Folding in Primate Cerebral Cortex.” Proceedings of the National Academy of Sciences 107, no. 44: 19008–19013. 10.1073/pnas.1012590107. PMC2973896 20956290

[hipo70119-bib-0034] Heuer, K. , N. Traut , L. Aristide , et al. 2025. “Principles of Neocortical Organisation and Behaviour in Primates.” bioRxiv. 10.1101/2025.07.17.665410.

[hipo70119-bib-0035] Huang, C. C. , E. T. Rolls , C. C. H. Hsu , J. Feng , and C. P. Lin . 2021. “Extensive Cortical Connectivity of the Human Hippocampal Memory System: Beyond the “What” and “Where” Dual Stream Model.” Cerebral Cortex 31, no. 10: 4652–4669. 10.1093/cercor/bhab113. 34013342 PMC8866812

[hipo70119-bib-0036] Igarashi, K. M. , L. Lu , L. L. Colgin , M.‐B. Moser , and E. I. Moser . 2014. “Coordination of Entorhinal–Hippocampal Ensemble Activity During Associative Learning.” Nature 510, no. 7503: 143–147. 10.1038/nature13162. 24739966

[hipo70119-bib-0037] Insausti, R. , D. G. Amaral , and W. M. Cowan . 1987. “The Entorhinal Cortex of the Monkey: II. Cortical Afferents.” Journal of Comparative Neurology 264, no. 3: 356–395. 10.1002/cne.902640306. 2445796

[hipo70119-bib-0039] Jeffery, K. J. , K. Cheng , N. S. Newcombe , V. P. Bingman , and R. Menzel . 2024. “Unpacking the Navigation Toolbox: Insights From Comparative Cognition.” Proceedings of the Royal Society B: Biological Sciences 291, no. 2016. 10.1098/rspb.2023.1304. PMC10846957 38320615

[hipo70119-bib-0040] Kaas, J. H. 1989. “Why Does the Brain Have So Many Visual Areas?” Journal of Cognitive Neuroscience 1, no. 2: 121–135. 10.1162/jocn.1989.1.2.121. 23968461

[hipo70119-bib-0041] Kaas, J. H. 2019. “The Origin and Evolution of Neocortex: From Early Mammals to Modern Humans.” Progress in Brain Research 250: 61–81. 10.1016/bs.pbr.2019.03.017. 31703909

[hipo70119-bib-0042] Kell, A. J. E. , S. L. Bokor , Y.‐N. Jeon , T. Toosi , and E. B. Issa . 2023. “Marmoset Core Visual Object Recognition Behavior Is Comparable to That of Macaques and Humans.” IScience 26, no. 1: 105788. 10.1016/j.isci.2022.105788. 36594035 PMC9804140

[hipo70119-bib-0043] Krettek, J. E. , and J. L. Price . 1977. “Projections From the Amygdaloid Complex and Adjacent Olfactory Structures to the Entorhinal Cortex and to the Subiculum in the Rat and Cat.” Journal of Comparative Neurology 172, no. 4: 723–752. 10.1002/cne.901720409. 838896

[hipo70119-bib-0044] Krubitzer, L. 2007. “The Magnificent Compromise: Cortical Field Evolution in Mammals.” Neuron 56, no. 2: 201–208. 10.1016/j.neuron.2007.10.002. 17964240

[hipo70119-bib-0045] Krubitzer, L. 2009. “In Search of a Unifying Theory of Complex Brain Evolution.” Annals of the New York Academy of Sciences 1156, no. 1: 44–67. 10.1111/j.1749-6632.2009.04421.x. 19338502 PMC2666944

[hipo70119-bib-0046] Krubitzer, L. , H. Künzle , and J. Kaas . 1997. “Organization of Sensory Cortex in a Madagascan Insectivore, the Tenrec ( *Echinops telfairi* ).” Journal of Comparative Neurology 379, no. 3: 399–414. 9067832

[hipo70119-bib-0047] Künzle, H. 2002. “Distribution of Perihippocampo–Hippocampal Projection Neurons in the Lesser Hedgehog Tenrec.” Neuroscience Research 44, no. 4: 405–419. 10.1016/S0168-0102(02)00158-X. 12445628

[hipo70119-bib-0048] Künzle, H. 2003. “Neocortical Connections With Perihippocampal and Periamygdalar Regions in the Hedgehog Tenrec.” Anatomy and Embryology 207, no. 4–5: 389–407. 10.1007/s00429-003-0356-z. 14615890

[hipo70119-bib-0049] Künzle, H. , and S. Radtke‐Schuller . 2000. “The Subrhinal Paleocortex in the Hedgehog Tenrec: A Multiarchitectonic Characterization and an Analysis of Its Connections With the Olfactory Bulb.” Anatomy and Embryology 202, no. 6: 491–506. 10.1007/s004290000137. 11131016

[hipo70119-bib-0050] Lavenex, P. , W. A. Suzuki , and D. G. Amaral . 2002. “Perirhinal and Parahippocampal Cortices of the Macaque Monkey: Projections to the Neocortex.” Journal of Comparative Neurology 447, no. 4: 394–420. 10.1002/cne.10243. 11992524

[hipo70119-bib-0051] Liewald, D. , R. Miller , N. Logothetis , H.‐J. Wagner , and A. Schüz . 2014. “Distribution of Axon Diameters in Cortical White Matter: An Electron‐Microscopic Study on Three Human Brains and a Macaque.” Biological Cybernetics 108, no. 5: 541–557. 10.1007/s00422-014-0626-2. 25142940 PMC4228120

[hipo70119-bib-0052] Liu, C. , C. C.‐C. Yen , D. Szczupak , F. Q. Ye , D. A. Leopold , and A. C. Silva . 2019. “Anatomical and Functional Investigation of the Marmoset Default Mode Network.” Nature Communications 10, no. 1: 1975. 10.1038/s41467-019-09813-7. PMC6488610 31036814

[hipo70119-bib-0053] Luppi, A. I. , F. E. Rosas , P. A. M. Mediano , D. K. Menon , and E. A. Stamatakis . 2024. “Information Decomposition and the Informational Architecture of the Brain.” Trends in Cognitive Sciences 28, no. 4: 352–368. 10.1016/j.tics.2023.11.005. 38199949

[hipo70119-bib-0054] Majka, P. , S. Bai , S. Bakola , et al. 2020. “Open Access Resource for Cellular‐Resolution Analyses of Corticocortical Connectivity in the Marmoset Monkey.” Nature Communications 11, no. 1: 1133. 10.1038/s41467-020-14858-0. PMC7048793 32111833

[hipo70119-bib-0055] Majka, P. , T. A. Chaplin , H. Yu , et al. 2016. “Towards a Comprehensive Atlas of Cortical Connections in a Primate Brain: Mapping Tracer Injection Studies of the Common Marmoset Into a Reference Digital Template.” Journal of Comparative Neurology 524, no. 11: 2161–2181. 10.1002/cne.24023. 27099164 PMC4892968

[hipo70119-bib-0056] Maller, J. J. , T. Welton , M. Middione , F. M. Callaghan , J. V. Rosenfeld , and S. M. Grieve . 2019. “Revealing the Hippocampal Connectome Through Super‐Resolution 1150‐Direction Diffusion MRI.” Scientific Reports 9, no. 1: 2418. 10.1038/s41598-018-37905-9. 30787303 PMC6382767

[hipo70119-bib-0057] Manns, J. R. , and H. Eichenbaum . 2006. “Evolution of Declarative Memory.” Hippocampus 16, no. 9: 795–808. 10.1002/hipo.20205. 16881079

[hipo70119-bib-0058] Martinez‐Trujillo, J. 2025. “Why Do Primates Have View Cells Instead of Place Cells?” Trends in Cognitive Sciences 29, no. 3: 226–229. 10.1016/j.tics.2024.12.007. 39765412

[hipo70119-bib-0059] Mesulam, M. 1998. “From Sensation to Cognition.” Brain 121, no. 6: 1013–1052. 10.1093/brain/121.6.1013. 9648540

[hipo70119-bib-0060] Mishkin, M. , and L. G. Ungerleider . 1982. “Contribution of Striate Inputs to the Visuospatial Functions of Parieto‐Preoccipital Cortex in Monkeys.” Behavioural Brain Research 6, no. 1: 57–77. 10.1016/0166-4328(82)90081-X. 7126325

[hipo70119-bib-0061] Mohedano‐Moriano, A. , P. Pro‐Sistiaga , M. M. Arroyo‐Jimenez , et al. 2007. “Topographical and Laminar Distribution of Cortical Input to the Monkey Entorhinal Cortex.” Journal of Anatomy 211, no. 2: 250–260. 10.1111/j.1469-7580.2007.00764.x. 17573826 PMC2375768

[hipo70119-bib-0062] Muñoz, M. , and R. Insausti . 2005. “Cortical Efferents of the Entorhinal Cortex and the Adjacent Parahippocampal Region in the Monkey ( *Macaca fascicularis* ).” European Journal of Neuroscience 22, no. 6: 1368–1388. 10.1111/j.1460-9568.2005.04299.x. 16190892

[hipo70119-bib-0063] Oakley, D. A. 1981. “Brain Mechanisms of Mammalian Memory.” British Medical Bulletin 37, no. 2: 175–180. 10.1093/oxfordjournals.bmb.a071697. 7032651

[hipo70119-bib-0064] O'Keefe, J. , and L. Nadel . 1978. The Hippocampus as a Cognitive Map. Oxford university press.

[hipo70119-bib-0065] Orban, G. A. , D. Van Essen , and W. Vanduffel . 2004. “Comparative Mapping of Higher Visual Areas in Monkeys and Humans.” Trends in Cognitive Sciences 8, no. 7: 315–324. 10.1016/j.tics.2004.05.009. 15242691

[hipo70119-bib-0066] Palmer, S. M. , and M. G. P. Rosa . 2006. “Quantitative Analysis of the Corticocortical Projections to the Middle Temporal Area in the Marmoset Monkey: Evolutionary and Functional Implications.” Cerebral Cortex 16, no. 9: 1361–1375. 10.1093/cercor/bhj078. 16292001

[hipo70119-bib-0067] Patzke, N. , M. A. Spocter , K. Æ. Karlsson , et al. 2015. “In Contrast to Many Other Mammals, Cetaceans Have Relatively Small Hippocampi That Appear to Lack Adult Neurogenesis.” Brain Structure and Function 220, no. 1: 361–383. 10.1007/s00429-013-0660-1. 24178679 PMC8734557

[hipo70119-bib-0068] Petersen, S. E. , B. A. Seitzman , S. M. Nelson , G. S. Wig , and E. M. Gordon . 2024. “Principles of Cortical Areas and Their Implications for Neuroimaging.” Neuron 112, no. 17: 2837–2853. 10.1016/J.NEURON.2024.05.008. 38834069 PMC13339775

[hipo70119-bib-0069] Quian Quiroga, R. 2023. “An Integrative View of Human Hippocampal Function: Differences With Other Species and Capacity Considerations.” Hippocampus 33, no. 5: 616–634. 10.1002/hipo.23527. 36965048

[hipo70119-bib-0070] Rathjen, S. , R. Engelmann , S. Struif , T. Kaulisch , D. Stiller , and S. Löwel . 2003. “The Growth of Cat Cerebral Cortex in Postnatal Life: A Magnetic Resonance Imaging Study.” European Journal of Neuroscience 18, no. 7: 1797–1806. 10.1046/j.1460-9568.2003.02909.x. 14622214

[hipo70119-bib-0071] Reardon, P. K. , J. Seidlitz , S. Vandekar , et al. 2018. “Normative Brain Size Variation and Brain Shape Diversity in Humans.” Science 360, no. 6394: 1222–1227. 10.1126/science.aar2578. 29853553 PMC7485526

[hipo70119-bib-0072] Reznik, D. , D. S. Margulies , M. P. Witter , and C. F. Doeller . 2024. “Evidence for Convergence of Distributed Cortical Processing in Band‐Like Functional Zones in Human Entorhinal Cortex.” Current Biology 34, no. 23: 5457–5469.e2. 10.1016/j.cub.2024.10.020. 39488200

[hipo70119-bib-0073] Reznik, D. , R. Trampel , N. Weiskopf , M. P. Witter , and C. F. Doeller . 2023. “Dissociating Distinct Cortical Networks Associated With Subregions of the Human Medial Temporal Lobe Using Precision Neuroimaging.” Neuron 111, no. 17: 2756–2772.e7. 10.1016/j.neuron.2023.05.029. 37390820

[hipo70119-bib-0075] Ringo, J. L. 1991. “Neuronal Interconnection as a Function of Brain Size.” Brain, Behavior and Evolution 38, no. 1: 1–6. 10.1159/000114375. 1657274

[hipo70119-bib-0099] Rolls, E. T. 1999. “Spatial View Cells and the Representation of Place in the Primate Hippocampus.” Hippocampus 9, no. 4: 467–480. 10.1002/(SICI)1098-1063(1999)9:4〈467::AID-HIPO13〉3.0.CO;2-F. 10495028

[hipo70119-bib-0076] Rosa, M. , and P. R. Manger . 2005. “Clarifying Homologies in the Mammalian Cerebral Cortex: The Case of the Third Visual Area (v3).” Clinical and Experimental Pharmacology and Physiology 32, no. 5–6: 327–339. 10.1111/j.1440-1681.2005.04192.x. 15854138

[hipo70119-bib-0077] Rosa, M. G. P. , J. G. M. Soares , T. A. Chaplin , et al. 2019. “Cortical Afferents of Area 10 in Cebus Monkeys: Implications for the Evolution of the Frontal Pole.” Cerebral Cortex 29, no. 4: 1473–1495. 10.1093/cercor/bhy044. 29697775 PMC6676977

[hipo70119-bib-0078] Saab, A. S. , I. D. Tzvetanova , and K.‐A. Nave . 2013. “The Role of Myelin and Oligodendrocytes in Axonal Energy Metabolism.” Current Opinion in Neurobiology 23, no. 6: 1065–1072. 10.1016/j.conb.2013.09.008. 24094633

[hipo70119-bib-0079] Scannell, J. , C. Blakemore , and M. Young . 1995. “Analysis of Connectivity in the Cat Cerebral Cortex.” Journal of Neuroscience 15, no. 2: 1463–1483. 10.1523/JNEUROSCI.15-02-01463.1995. 7869111 PMC6577853

[hipo70119-bib-0080] Schroeder, C. E. , and J. Foxe . 2005. “Multisensory Contributions to Low‐Level, ‘Unisensory’ Processing.” Current Opinion in Neurobiology 15, no. 4: 454–458. 10.1016/j.conb.2005.06.008. 16019202

[hipo70119-bib-0081] Seltzer, B. , and D. N. Pandya . 1984. “Further Observations on Parieto‐Temporal Connections in the Rhesus Monkey.” Experimental Brain Research 55, no. 2: 301–312. 10.1007/BF00237280. 6745368

[hipo70119-bib-0082] Smaers, J. B. , R. S. Rothman , D. R. Hudson , et al. 2021. “The Evolution of Mammalian Brain Size.” Science Advances 7, no. 18: 9. 10.1126/sciadv.abe2101. PMC8081360 33910907

[hipo70119-bib-0083] Smiley, J. F. , T. A. Hackett , I. Ulbert , et al. 2007. “Multisensory Convergence in Auditory Cortex, I. Cortical Connections of the Caudal Superior Temporal Plane in Macaque Monkeys.” Journal of Comparative Neurology 502, no. 6: 894–923. 10.1002/cne.21325. 17447261

[hipo70119-bib-0084] Solomon, S. G. , and M. G. P. Rosa . 2014. “A Simpler Primate Brain: The Visual System of the Marmoset Monkey.” Frontiers in Neural Circuits 8, no. AUG: 96. 10.3389/fncir.2014.00096. 25152716 PMC4126041

[hipo70119-bib-0085] Stevens, C. F. 1989. “How Cortical Interconnectedness Varies With Network Size.” Neural Computation 1, no. 4: 473–479. 10.1162/neco.1989.1.4.473.

[hipo70119-bib-0086] Suárez, R. , A. Paolino , L. R. Fenlon , et al. 2018. “A Pan‐Mammalian Map of Interhemispheric Brain Connections Predates the Evolution of the Corpus Callosum.” Proceedings of the National Academy of Sciences 115, no. 38: 9622–9627. 10.1073/pnas.1808262115. PMC6156618 30181276

[hipo70119-bib-0087] Suzuki, W. L. , and D. G. Amaral . 1994. “Perirhinal and Parahippocampal Cortices of the Macaque Monkey: Cortical Afferents.” Journal of Comparative Neurology 350, no. 4: 497–533. 10.1002/cne.903500402. 7890828

[hipo70119-bib-0089] Tolman, E. C. 1948. “Cognitive Maps in Rats and Men.” Psychological Review 55, no. 4: 189–208. 10.1037/h0061626. 18870876

[hipo70119-bib-0090] Tulving, E. 1985. “How Many Memory Systems Are There?” American Psychologist 40, no. 4: 385–398. 10.1037/0003-066X.40.4.385.

[hipo70119-bib-0091] Uylings, H. B. M. , H. J. Groenewegen , and B. Kolb . 2003. “Do Rats Have a Prefrontal Cortex?” Behavioural Brain Research 146, no. 1–2: 3–17. 10.1016/j.bbr.2003.09.028. 14643455

[hipo70119-bib-0100] Ventura‐Antunes, L. , B. Mota , and S. Herculano‐Houzel . 2013. “Different Scaling of White Matter Volume, Cortical Connectivity, and Gyrification Across Rodent and Primate Brains.” Frontiers in Neuroanatomy 7: 3. 23576961 10.3389/fnana.2013.00003 PMC3620553

[hipo70119-bib-0092] Vincent, J. L. , I. Kahn , D. C. Van Essen , and R. L. Buckner . 2010. “Functional Connectivity of the Macaque Posterior Parahippocampal Cortex.” Journal of Neurophysiology 103, no. 2: 793–800. 10.1152/jn.00546.2009. 19955295 PMC2822694

[hipo70119-bib-0093] Vincent, J. L. , G. H. Patel , M. D. Fox , et al. 2007. “Intrinsic Functional Architecture in the Anaesthetized Monkey Brain.” Nature 447, no. 7140: 83–86. 10.1038/nature05758. 17476267

[hipo70119-bib-0094] Wang, S.‐F. , M. Ritchey , L. A. Libby , and C. Ranganath . 2016. “Functional Connectivity Based Parcellation of the Human Medial Temporal Lobe.” Neurobiology of Learning and Memory 134: 123–134. 10.1016/j.nlm.2016.01.005. 26805590 PMC4955645

[hipo70119-bib-0095] Wang, X.‐J. , U. Pereira , M. G. Rosa , and H. Kennedy . 2020. “Brain Connectomes Come of Age.” Current Opinion in Neurobiology 65: 152–161. 10.1016/j.conb.2020.11.002. 33276230 PMC7770070

[hipo70119-bib-0096] Witter, M. P. , H. J. Groenewegen , F. H. Lopes da Silva , and A. H. M. Lohman . 1989. “Functional Organization of the Extrinsic and Intrinsic Circuitry of the Parahippocampal Region.” Progress in Neurobiology 33, no. 3: 161–253. 10.1016/0301-0082(89)90009-9. 2682783

[hipo70119-bib-0088] Yeo, B. T. , F. M. Krienen , J. Sepulcre , et al. 2011. “The Organization of the Human Cerebral Cortex Estimated by Intrinsic Functional Connectivity.” Journal of Neurophysiology 106, no. 3: 1125–1165. 10.1152/jn.00338.2011. 21653723 PMC3174820

[hipo70119-bib-0097] Zheng, A. , D. F. Montez , S. Marek , et al. 2021. “Parallel Hippocampal‐Parietal Circuits for Self‐ and Goal‐Oriented Processing.” Proceedings of the National Academy of Sciences 118, no. 34: e2101743118. 10.1073/pnas.2101743118. PMC8403906 34404728

[hipo70119-bib-0098] Zhu, S. L. , K. J. Lakshminarasimhan , and D. E. Angelaki . 2023. “Computational Cross‐Species Views of the Hippocampal Formation.” Hippocampus 33, no. 5: 586–599. 10.1002/hipo.23535. 37038890 PMC10947336

